# Gender and Social Stratification in Active Aging: Inequalities in Sport Participation and Subjective Health Among Older Adults in South Korea

**DOI:** 10.3390/healthcare13233124

**Published:** 2025-12-01

**Authors:** Su Yeon Roh, Ik Young Chang

**Affiliations:** 1 Department of Exercise Rehabilitation, College of Medical Science, Gachon University, Inchon 21936, Republic of Korea; dr.rohpilates@gachon.ac.kr; 2 Department of Sport Coaching, College of Sport Science, Korea National Sport University, Seoul 05541, Republic of Korea

**Keywords:** sport participation, aging, gender, social stratification, health, older adults, South Korea

## Abstract

**Background**: As South Korea transitions into a super-aged society, promoting sport participation among older adults is increasingly vital for physical health, emotional well-being, and social inclusion. **Objective**: This study examines how the interplay between gender and social stratification influence sport participation and health among South Koreans aged 60 and above. **Methods**: Using data from the 2024 Korea National Sports Participation Survey (*n* = 1779), this study employed Multiple Correspondence Analysis (MCA), cross-tabulation, and one-way ANOVA with Scheffé’s post hoc tests to examine differences in sport participation and health by gender and social stratification such as income, education, and occupation. **Results**: The analysis revealed significant differences in sport participation and subjective health outcomes by gender and social stratification. Among older men, sport participation varied strongly by socioeconomic status: higher-status men participated in golf, cycling, and bodybuilding, whereas those from lower strata mainly engaged in walking and gateball. In contrast, older women’s participation types were less stratified and more influenced by gender norms, with consistent involvement in walking, aerobics, yoga, and stretching. One-way ANOVA showed statistically significant differences (*p* < 0.001) in subjective health status and physical fitness by all socioeconomic variables for both genders. **Conclusions**: Older adults’ sport participation and health in South Korea are constrained by both socioeconomic inequality and entrenched gender norms. Promoting equitable active aging requires policies that both reduce socioeconomic barriers and challenge restrictive gender norms.

## 1. Introduction

South Korea is undergoing one of the fastest demographic transitions toward a super-aged society worldwide, with projections indicating that more than 20% of the population will be over 65 by 2025 [1]. This rapid demographic shift has generated multidimensional social challenges, including health inequalities, social isolation, and intergenerational tensions. Within this context, sports participation functions as more than a leisure activity; it represents an important mechanism for promoting physical health, psychological well-being, and social integration among older adults. Nevertheless, opportunities for sports participation are not distributed equally. Socioeconomic status and gender operate as critical determinants, producing structural inequalities that shape the accessibility, type, and frequency of sports engagement.

A substantial body of literature has examined the relationship between social stratification and sports participation in South Korea. Analyses of national survey data have shown that individuals of higher socioeconomic status are more likely to participate in capital-intensive and symbolically prestigious sports such as golf and tennis, typically in private or workplace facilities, whereas those of lower socioeconomic status concentrate on low-cost activities such as walking, gateball, or stretching exercises in public facilities [2]. Such findings highlight how sport operates as a space where social distinctions are both displayed and perpetuated through unequal distributions of economic and cultural capital [3,4]. Similar arguments have been advanced in international contexts, where sport is seen as reflecting broader patterns of social inequality [5] and where socioeconomic status significantly influences patterns of regular participation [6].

Gender inequality constitutes another crucial axis of stratification in sports participation and health. Research indicates that Korean men tend to engage in capital-intensive and competitive sports, while women are disproportionately concentrated in activities such as aerobics, yoga, and dance sports, which have historically been framed as “feminine sports” [7]. This pattern is consistent with evidence that gender stereotypes and role expectations act as significant constraints on both participation and performance in sport [8].

Building on these insights, prior studies have demonstrated that the intersection of gender and social stratification produces compounded disadvantages in sports contexts by reinforcing structural barriers and cumulative inequalities [9]. In this sense, sports should not be understood as neutral spaces of leisure but rather as fields in which gendered and class-based inequalities are continually reproduced. Nevertheless, most existing studies conceptualize adults aged 18 and over as a single, homogeneous group, thereby overlooking populations that require more concentrated analytical attention.

In particular, sports participation is closely tied to inequalities in health, making them especially consequential for older adults experiencing advanced stages of aging. This population often faces intersecting challenges such as declining physical capacity, accumulated chronic conditions, and heightened risks of social isolation [10]. Consequently, gender- and class-based disparities in sports participation can have direct and detrimental effects on their health and physical functioning. For these reasons, it is both necessary and appropriate to examine inequalities in sports participation—and their implications for subjective health—with a specific focus on older adults.

Therefore, this research aims to examine gender- and social stratification-based inequalities in sports participation and health among older adults in South Korea. Specifically, it addresses two research questions: (1) Are there differences in types of sports participation according to gender and social stratification among older adults in South Korea? (2) Are there differences in subjective health according to social stratification by gender among older adults in South Korea?

In the context of South Korea’s rapidly advancing super-aged society, addressing inequalities in sports participation and health is an urgent social priority with implications for public health, welfare, and social integration. Integrating age as an analytical dimension alongside social stratification and gender thus contributes to a more comprehensive and intersectional understanding of how inequalities are reproduced in sports [2,3,6,7,11,12], and provides essential empirical foundations for developing gender- and stratification-sensitive policies that promote equitable sports opportunities for older adults.

## 2. Materials and Methods

This study utilized data from the 2024 Korea National Sports Participation Survey, an annual survey on participation in physical activities conducted by the Korean Ministry of Culture, Sports and Tourism [13]. The survey targeted Korean citizens aged 10 years or older, with a total sample of 9000 individuals (specifically, one person from each of 9000 households).

In South Korea, the Elderly Welfare Act defines an “older adult” as an individual aged 65 or older. However, this study adopts age 60 as the analytical threshold, reflecting its legal and institutional relevance as the statutory retirement age across most sectors. Retirement at age 60 typically marks a withdrawal from the labor market, and its implications for sports participation are the subject of ongoing scholarly debate. Some studies suggest that increased discretionary time after retirement facilitates greater engagement in sports [14], whereas others highlight the constraining effects of reduced income on participation [15]. From both a life-course and social stratification perspective, the transition at age 60 constitutes a critical juncture at which notable shifts in sports participation types may occur. Accordingly, including individuals aged 60 and above enables this study to capture the early onset of structural inequalities associated with retirement and aging in the South Korean context.

From the raw dataset, 6371 individuals under the age of 60 were excluded, resulting in 2629 respondents aged 60 and above. Of these, 738 reported not participating regularly in any physical activities and were therefore excluded from the analysis. Additionally, frequency analysis was conducted to exclude 112 participants engaged in sports with fewer than 30 regular participants (as identified through frequency analysis).

Consequently, a total of 1779 respondents aged 60 years and older who reported regular participation in physical activities were included in the analysis. This survey was approved by the Ethics Committee of the Korean Ministry of Culture, Sports and Tourism (approval code: 113003).

### 2.1. Variables

In order to explore differences in types of sports participation according to gender and social stratification among Korean older adults, this study set gender, monthly household income level, educational background, and occupation as variables. Social stratification was examined using three variables: monthly household income, educational background, and occupation. Specifically, monthly household income levels were classified into four groups: less than 2055 USD; 2055 USD to less than 2740 USD; 2740 USD to less than 3425 USD; and 3425 USD and above. Educational background was classified into three groups: up to lower secondary; upper secondary; and bachelor’s level or higher. Finally, occupations were classified into four groups: no occupation (homemaker or unemployed); blue-collar workers; sales and service workers; office and professionals.

The classification of each variable was redefined in accordance with the objectives of this study. These classifications took into account Korea’s average monthly household income in 2024, as reported by Statistics Korea [16]; compulsory elementary and middle school education; high school and college entrance rates; occupational categories and frequencies; and the Korean Standard Classification of Occupations [17]. In addition, regarding sports events, 10 sports with 30 or more regular participants, among those specified in the 2024 KNSPS, were designated as dependent variables [13].

In order to examine differences in health status according to social stratification by gender, social stratification such as income, education and occupation was treated as the independent variable, while subjective health status and subjective physical fitness, which were measured using a five-point Likert scale, were established as dependent variables for analysis. Table 1 presents demographic information about the participants.

### 2.2. Data Analysis

This study employed SPSS 26.0 to analyze the data. Multiple correspondence analysis (MCA) and cross-tabulation analysis (cross-tab) were conducted to examine differences in types of sports participation. MCA is a statistical analysis technique used to visualize the row and column information of a contingency table in a two-dimensional figure and to classify respondents with similar and dissimilar patterns according to their survey responses. More specifically, MCA analyzes the correlation between variables by calculating the distance between two or more categorical variables and visually presenting the results [18]. This study examines how to show the correlation of sports participation according to gender and social stratification among older adults by using the MCA method designed to calculate the distance in a two-dimensional space.

A cross-tab was conducted to examine differences in sports participation according to gender and social stratification among older Korean adults. This analysis is particularly significant not only for identifying differences in frequency through the cross-tab, but also in setting the range of correlation between variables shown through the MCA.

An adjusted residual (AR) greater than 1.96 indicates that the number of cases in that cell is significantly larger than expected at the 0.05 significance level, whereas an AR less than –1.96 indicates that the number of cases is significantly smaller than expected. If there is a statistically significant difference between groups based on MCA and AR values, researchers should pay attention to the groups in which the absolute value of the AR exceeds 1.96, as these indicate meaningful deviations from the expected patterns.

For instance, if the cycling participation ARs for male and female groups with incomes of 2055 USD to less than 2740 USD are +3.5 and –1.4, respectively, it can be interpreted that men in this income group actively participated in cycling, whereas women did not show a statistically meaningful deviation from the expected value.

In addition, a one-way analysis of variance (ANOVA) was performed to identify statistically significant differences in subjective health status and subjective physical fitness according to social stratification variables such as income level, educational background, and occupation. When the ANOVA results indicated statistically significant differences among groups, Scheffé’s post hoc test was subsequently applied to determine the specific sources of variation. Scheffé’s test was selected because it is a conservative procedure that performs robustly with unequal group sizes, which was important for this study since social stratification variables naturally create imbalanced group distributions, and because it supports both pairwise and more complex multiple comparisons. Statistical significance was set at *p* < 0.05.

## 3. Results

### 3.1. Differences in Sports Participation Types by Gender and Social Stratification Among Older Adults

In the Results section, Multiple Correspondence Analysis (MCA) and cross-tabulation were conducted to examine differences in sport participation types according to gender and social stratification. Additional explanations are provided below to facilitate an understanding of the MCA outcomes.

The model extracted two dimensions with eigenvalues greater than 1, which together accounted for 68.17% of the total variance (Table 2).

**Figure 1:**
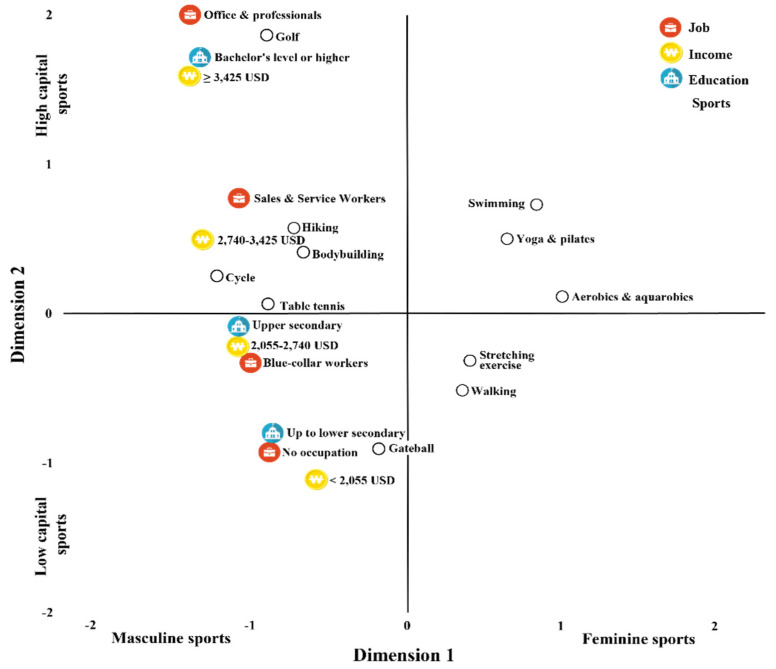
Results of MCA for types of sports participation according to the social stratification of older men.

**Figure 2:**
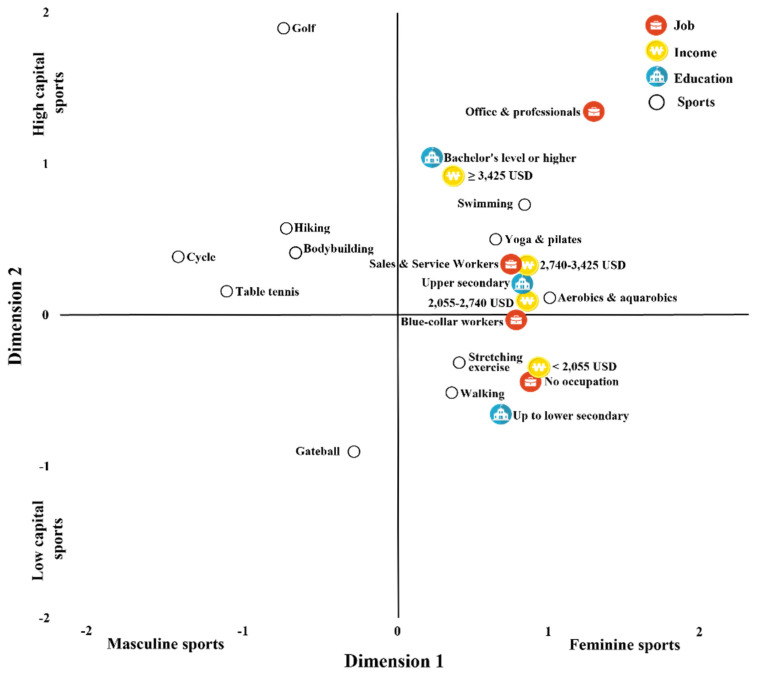
Results of MCA for types of sports participation according to the social stratification of older women.

Although a single MCA was performed including all categories (sport type, education level, occupation, and income level), the results are presented in two separate plots (Figure 1 and Figure 2) to visually compare the spatial distribution of categories by gender. These two plots reflect the same analytical model but are divided into male and female respondent groups to enhance interpretability and improve visual clarity.

#### 3.1.1. MCA of Types of Sports Participation by Gender and Social Stratification Among Older Adults

In this study, the results of the MCA are presented in a two-dimensional representation (See Figure 1 and Figure 2). For interpretive clarity, the axes are labeled using the concepts “Masculine Sports” and “Feminine Sports,” as well as “Low-Capital Sports” and “High-Capital Sports.” These labels do not reflect arbitrary researcher-driven interpretations; rather, they align with well-established patterns documented in previous research.

First, sports have historically been categorized as “masculine” or “feminine” based on culturally embedded stereotypes related to physical contact, competitiveness, esthetic emphasis, and bodily expressiveness. Masculine sports are typically associated with strength, aggression, and direct physical confrontation (e.g., American football, rugby, baseball, taekwondo), whereas feminine sports are characterized by grace, esthetics, and expressiveness (e.g., aerobics, yoga, dance sports, artistic swimming). A substantial body of literature in Korea and internationally confirms that these gendered perceptions shape individuals’ participation choices and opportunities [8,19,20,21,22,23].

Second, numerous studies have shown systematic class-based differentiation in sports participation. Individuals in higher socioeconomic strata tend to participate in capital-intensive and symbolically prestigious sports such as golf, tennis, and skiing, whereas those in lower socioeconomic strata more commonly engage in low-cost or widely accessible activities such as walking, aerobics, soccer, or ice hockey [6,24,25,26,27]. These distinctions reflect how economic and cultural capital structure access to particular types of sports and contribute to socially stratified types of participation.

Figure 1 presents a two-dimensional representation of the MCA results on types of sports participation among older Korean men according to their social stratification.

When older men’s social stratification and their types of sports participation were grouped according to the distance features shown in the figure, older men with incomes of less than 2055 USD, those with an educational background up to lower secondary, and those with no occupation (homemaker or unemployed) showed a tendency to participate in gateball.

In comparison, those with incomes of 2055 USD to less than 2740 USD or 2740 USD to less than 3425 USD, those with an upper secondary educational background, and those employed as blue-collar or sales and service workers tended to participate in table tennis, hiking, bodybuilding, and cycling.

Finally, those with incomes of 3425 USD and above, a bachelor’s degree or higher, and employment in office or professional occupations clearly showed a tendency to participate in golf.

Figure 2 presents a two-dimensional representation of the MCA results on types of sports participation among older Korean women according to their social stratification.

When older women’s social stratification and their types of sports participation were grouped according to the distance features shown in the figure, those with incomes of less than 2055 USD, those with up to lower secondary education, and those with no occupation showed a tendency to participate in walking and stretching exercises.

In contrast, those with incomes of 2055 USD to less than 2740 USD or 2740 USD to less than 3425 USD, those with an upper secondary educational background, and those employed as blue-collar or sales and service workers tended to participate in yoga and Pilates, aerobics, and aquarobics.

Finally, those with incomes of 3425 USD and above, those with a bachelor’s degree or higher, and those employed in office or professional occupations showed a tendency to participate in swimming.

#### 3.1.2. Cross-Tab of Types of Sports Participation by Gender and Social Stratification Among Older Adults

This study conducted a cross-tab to provide a better understanding of the two-dimensional schema shown in the MCA results on types of sports participation according to gender and social stratification among older adults. Table 3 shows the cross-tab results of sports participation according to the social stratification of older men and women. Men with an income of less than 2055 USD participated in walking (4.2), gateball (2.8), while women with the same income participated in walking (5.0), aerobics & aquarobics (2.5), yoga & pilates (4.3), stretching exercise (3.4). In contrast, men with an income of 2055 USD to less than 2740 USD participated in hiking (2.6), cycle (3.5), table tennis (5.0), while women with the same income range participated in swimming (4.3). Men with an income of 2740 USD to less than 3425 USD participated in golf (3.3), hiking (3.7), bodybuilding (2.6), while women with the same income range participated in aerobics & aquarobics (4.1), swimming (2.1), yoga & pilates (2.6). Finally, men with an income of 3425 USD and above participated in golf (9.8), hiking (7.2), bodybuilding (2.6), cycle (3.6), while women with the same income or above participated in hiking (2.1), swimming (3.0).

The types of sports that older men and women participated in were examined according to their educational backgrounds. Men with an educational background of up to lower secondary school participated in walking (3.9), table tennis (2.1), while women with the same educational participated in walking (4.5), yoga & pilates (5.0), stretching exercise (4.3). Men with an educational background of upper secondary school participated in hiking (9.5), bodybuilding (3.1), cycle (4.7), table tennis (3.3), while women with the same educational background participated in aerobics & aquarobics (5.0), and swimming (4.6). Finally, men with an educational background of bachelor’s level or higher participated in golf (13.7), bodybuilding (2.6), cycle (2.0), table tennis (2.2), while women with the same educational background participated in golf (3.1), swimming (3.6), and yoga & pilates (5.4).

Sports participation among older men and women was also examined according to their occupations. Clear distinctions between men and women were observed. Among women, only those with no occupation participated in walking (2.7), aerobics & aquarobics (5.9), swimming (4.6), and yoga & pilates (5.3). In contrast, men with no occupation participated in walking (4.0) and gateball (4.3). Blue-collar workers participated in hiking (5.7), cycle (4.8), and table tennis (2.6). Men in service & sales occupations participated in golf (4.7), hiking (5.3), bodybuilding (3.4), cycling (3.3), and table tennis (5.3). Finally, men categorized as office workers & professional occupations participated in golf (12.8), hiking (2.6), and bodybuilding (2.0).

The findings of this study indicate that the types of sports in which older men and women participate are clearly distinguished by income, educational background, and occupation, which are key factors of social stratification. Men’s participation in sports reflects the stratification of their income, education, and occupation. For example, upper-class men tend to participate in golf, hiking, and fitness-related activities such as bodybuilding and cycle, while lower-class men are more likely to participate in gateball, walking. In contrast, women’s sports participation is not strongly differentiated by social stratification but is instead concentrated within a relatively narrow range of activities. Women mainly participate in walking, swimming, yoga & pilates, aerobics & aquarobics, stretching exercises relatively regardless of their income, education, or occupation in comparison with men’s sports participation.

### 3.2. Differences in Health Status by Gender and Social Stratification Among Older Adults

This study conducted a one-way ANOVA to examine differences in subjective health status and subjective physical fitness according to social stratification by gender among older adults in South Korea.

In Table 4, this result indicated significant differences in subjective health status according to income, education, and occupation among both men and women older adults (*p* < 0.001). More specifically, among men, those with higher income levels reported significantly higher subjective health status (F (3, 830) = 31.739, *p* < 0.001). Scheffé’s post hoc analysis revealed that men earning less than 2055 USD per month scored significantly lower than those in all higher income groups. Similarly, educational attainment showed a clear gradient effect (F (2, 831) = 50.147, *p* < 0.001), where subjective health improved sequentially from the lower-secondary group to the upper-secondary and bachelor’s or higher groups (a < b < c). Occupation also displayed significant disparities (F (3, 830) = 43.379, *p* < 0.001), with inactive men reporting the lowest subjective health compared to blue-collar, sales & service, and office & professionals.

Among women, subjective health status similarly varied across income (F (3, 941) = 46.094, *p* < 0.001), education (F (2, 942) = 50.624, *p* < 0.001), and occupation (F (3, 942) = 13.376, *p* < 0.001). Post hoc comparisons indicated that women with lower income (less than 2055 USD) reported significantly lower subjective health than those in middle- and higher-income groups. Those with upper-secondary or higher education demonstrated higher subjective health than women with only lower-secondary education. In terms of occupation, inactive women showed significantly lower subjective health than those engaged in sales or service positions.

Overall, these findings demonstrate that subjective health status increases consistently with higher socioeconomic status across both genders.

As shown in Table 5, significant differences were shown in subjective physical fitness across income, education, and occupational groups among both men and women older adults (*p* < 0.001).

Among men, higher income levels were associated with significantly greater subjective physical fitness (F (3, 830) = 34.565, *p* < 0.001). Scheffé’s post hoc analysis indicated that participants earning less than 2055 USD per month scored significantly lower than those in all higher income groups. Educational background demonstrated a similar pattern, (F (2, 831) = 42.578, *p* < 0.001), with men holding a bachelor’s degree or higher reporting significantly higher subjective physical fitness than those with lower educational levels (a < b < c). Significant occupational differences were also identified, (F (3, 830) = 46.873, *p* < 0.001), showing that inactive men perceived their physical fitness as significantly lower than blue-collar, sales/service, and professional workers (a < b, c, d).

Among women, comparable patterns were observed. Subjective physical fitness differed significantly by income (F (3, 941) = 28.833, *p* < 0.001), education (F (2, 942) = 49.014, *p* < 0.001), and occupation (F (3, 942) = 12.893, *p* < 0.001). Scheffé’s test revealed that women with higher income and educational levels reported significantly greater subjective physical fitness, while inactive women showed lower fitness perceptions compared with those employed in sales and service occupations.

In sum, these results demonstrate that socioeconomic status is a robust and consistent predictor of subjective physical fitness among older adults in South Korea. Individuals with higher levels of income, education, and occupational status reported substantially more positive self-assessments of physical fitness, highlighting the persistent structural inequalities rooted in social stratification.

## 4. Discussion

Previous studies have consistently confirmed that variables such as gender and social stratification play a critical role in determining the types of sports in which individuals participate [2,7,26,27,28,29,30,31]. For older adults, sport participation is particularly significant as it is directly connected to both physical and mental health. Therefore, inequalities in the choice of sports among older adults should not be regarded merely as matters of personal preference, but rather as potential constraints that may be associated with differences in health and quality of life. Against this backdrop, the present study examines how older men and women differ in their types of sport participation across gender and social stratification, and this section discusses and interprets the findings.

The findings revealed that older men primarily participated in walking, gateball, golf, hiking, bodybuilding, cycling, and table tennis, whereas older women engaged primarily in walking, aerobics and aqua aerobics, swimming, yoga and Pilates, and stretching exercises. These differences are closely connected to the tendency in Korean society to construct certain sports as masculine or feminine. The gendered perception of sports has been consistently documented across decades of research [7,32,33]. Such studies have demonstrated that men are more likely to participate in sports that emphasize attributes such as risk, speed, strength, team spirit, and aggressiveness [34,35]. Specifically, these include rugby, soccer, combat sports, cycling, and other team sports that require physical contact, strength, and the use of heavy equipment. By contrast, women are more associated with characteristics such as elegance, expressiveness, and flexibility, focusing on sports that emphasize esthetic elements and harmony, including dance, figure skating, gymnastics, aerobics, swimming, Pilates, and stretching [34,35,36,37].

In particular, this study found that the distinction between masculine and feminine sports was more pronounced among older adults compared to the results reported by Roh & Chang [7] for the general adult population. While Roh & Chang [7] noted that men also participated in stretching exercises, none of the older men in this study engaged in stretching, which was predominantly practiced by older women. The participants in this study were born before 1965, a generation that grew up in a social context in Korea characterized by rigid gender roles and norms [38,39]. This historical background has influenced types of sport participation, producing a clear gender-based division in the choice and manner of participation [26].

Along with gender, social stratification is another factor that influences participation in specific sports. Roh and Chang [7] observed that among men, participation was distinctly shaped by social stratification, whereas for women, the availability of time resources proved to be a more critical determinant than social stratification. The present study confirmed similar results.

Specifically, sport participation among older women appeared relatively unaffected by social stratification. Differences were observed only in a few cases: golf was practiced exclusively by women with at least a university degree, while stretching exercises were performed exclusively by women with monthly incomes less than 2055 USD or with an education level of middle school or lower. However, aerobics and aqua aerobics, yoga and Pilates, and swimming showed relatively equal participation regardless of social stratification. This finding is consistent with the results of Roh & Chang [7]. Previous studies have explained that middle-aged women widely prefer activities such as aqua aerobics and aerobics because they carry lower risks of injury compared to other sports, contribute to body management and body-image improvement, and enhance positive emotions. Furthermore, since these programs are generally offered at relatively low cost in welfare centers, cultural centers, and public sports facilities in Korea, they are perceived as public sports accessible to all. This helps explain the results of the present study, where participation opportunities were broadly available regardless of differences in social stratification [40].

In addition, this study revealed that older women’s sport participation was closely associated with the availability of time resources. Women with no occupation participated broadly across the five categories examined, whereas women who were employed showed extremely limited participation. In this regard, Breuer et al. [26] reported that sport participation among middle-aged women differed according to working hours. Specifically, women with long working hours were more likely to prefer fitness or dance activities rather than walking. This suggests that for women, sport participation appears to be more closely associated with the availability of time resources than by economic status.

By contrast, the sport participation of older men was clearly differentiated by social stratification. Their participation could be categorized into four groups: (1) walking and gateball, which lower-class groups mainly practiced; (2) hiking and cycling, which lower-class groups rarely practiced; (3) golf and bodybuilding, which were practiced exclusively by higher-class groups; and (4) table tennis, which was accessible regardless of social stratification.

Gateball is considered a representative senior sport, mainly practiced by older men with low income or no occupation. This is because gateball is often provided as part of welfare center programs for the elderly, carries little to no financial burden, and is perceived as appropriate due to its low level of physical intensity [41]. Jin & Chang [2], who analyzed sport participation by social stratification, also found that gateball participation was concentrated among older adults in lower social strata, reflecting the structural constraints faced by economically vulnerable seniors. By contrast, hiking and cycling require certain expenses for equipment purchase and maintenance, while golf and bodybuilding demand even higher levels of ongoing costs and investment (e.g., memberships, lessons, equipment). Thus, these activities are primarily practiced by older adults in higher social strata [42]. In comparison, table tennis has high accessibility and low cost, making it a sport that can be practiced regardless of social strata [2].

An interesting finding is that, unlike older women, older men with no occupation were less engaged in sport participation. In other words, having more time resources did not necessarily translate into greater participation. Why, then, did such contrasting results emerge between older men and women? Why was the range of sports practiced by older men with no occupation so limited? This can be explained by the fact that retirement for women often serves as an opportunity to expand leisure resources, whereas for men it is not merely the loss of employment a factor that has been linked in prior research to identity challenges and reduced social relationships [43].

In Korean society, women have traditionally structured their daily lives around both family and work, whereas men have organized their lives primarily around employment [43]. Because men’s employment was taken for granted while women’s employment was regarded as optional, men’s unemployment severely undermined their identity as household heads and primary breadwinners, whereas the impact of women’s unemployment was relatively weaker [44,45]. Numerous studies have also reported that retired older men experience a reduction in social roles, loss of confidence, and feelings of helplessness, and are more likely than older women to suffer from depression [46,47,48]. In this context, the lower level of sport participation among older men with no occupation, compared to older women with no occupation, should not be seen merely as a matter of leisure activity but as directly related to the structural challenges men face in reconstructing identity and maintaining social engagement after retirement.

In addition, for older adults, low-intensity rather than high-intensity exercise generally has positive physical and emotional effects. Specifically, in old age, low-intensity physical activity improves joint and bone health, enhances physical and emotional functioning, and is naturally preferred because it is safe, carries a lower risk of injury, and is relatively cost-efficient. As a result, older adults tend to favor low-impact sports such as aqua aerobics, yoga, stretching, and swimming [49]. However, many of these low-impact sports are socially perceived as women’s sports, creating difficulties for older men to participate due to gender norms. Consequently, older men with no occupation tend to choose activities that are less constrained by gender norms, pose a low risk of injury, and entail low cost—namely, walking and gateball. This limits their sport participation and acts as a barrier to expanding participation into more diverse sports. Importantly, gender norms in sport do not only restrict participation options but also affect psychological well-being [50]. These results suggest that sport participation, particularly when individuals can overcome or navigate restrictive gender norms, has been associated with better mental health outcomes in older age. Thus, beyond the physical and social benefits, sport engagement may play a crucial role in supporting the emotional and psychological well-being of older adults, especially in the face of normative gendered expectations.

The study also examined gender- and social stratification-based differences in subjective health status and subjective physical fitness among older adults in South Korea, revealing a consistent gradient according to socioeconomic status. Across both men and women, individuals with higher levels of income, education, and occupational status reported significantly higher subjective health status and physical fitness. These findings highlight that socioeconomic status and social stratification show strong associations with health inequality in later life, even within a highly developed welfare and healthcare context such as South Korea.

The results align with a substantial body of international evidence demonstrating that social stratification is strongly associated with physical and psychological well-being in old age. Marmot and Wilkinson [51] argued that the social gradient in health persists across societies because individuals with higher socioeconomic resources experience greater control over their lives and environments, leading to healthier behaviors and lower stress exposure. The patterns observed in this study suggest that sport participation may be part of the broader behavioral patterns related to the reproduction of these inequalities among older adults in South Korea.

From a sociological perspective, these results also resonate with Bourdieu’s theory of social reproduction, which posits that individuals’ access to sports and health-promoting activities reflects their accumulated economic, cultural, and social capital [3]. Individuals situated higher in the social hierarchy often possess the financial resources, education, and social networks necessary to participate in structured and health-oriented physical activities, whereas those with lower positions in the social stratification system face barriers related to cost, accessibility, and time. These inequalities are further compounded by gendered social roles and cultural norms that limit older women’s opportunities for active leisure and reinforce passive or caregiving roles within families [52].

Gender differences observed in this study are consistent with findings from other societies. For instance, Lee et al. [53] demonstrated that physical activity significantly reduces mortality risk among older adults, but its protective effects are moderated by socioeconomic status, highlighting the persistent role of social inequality in shaping health outcomes. In South Korea, traditional gender norms and post-retirement economic dependence may intensify such disparities, especially among women from lower socioeconomic backgrounds. Accordingly, targeted policy interventions are necessary to reduce these socioeconomic disparities and ensure equitable health opportunities for older adults.

## 5. Conclusions and Limitations

The results of this study highlight clear gender- and social stratification-based differences in sport participation among older adults in South Korea. Older men primarily participated in walking, gateball, golf, hiking, bodybuilding, cycling, and table tennis, while older women mainly engaged in walking, aerobics and aqua aerobics, swimming, yoga and Pilates, and stretching exercises. Among older men, participation types were strongly influenced by socioeconomic status, with those without paid occupations being limited almost exclusively to walking and gateball. In contrast, older women showed relatively little variation across social strata. Those not engaged in paid work participated in a broader range of low-impact activities, suggesting that increased time availability facilitates more diverse sport participation. Moreover, socioeconomic status in both men and women older adults in South Korea emerged as a robust and consistent predictor of subjective health status and subjective physical fitness. Individuals with higher levels of income, education, and occupational status reported significantly more positive self-assessments, underscoring the persistent structural inequalities rooted in social stratification.

This study contributes to the existing literature by examining gendered and socially stratified differences in sport participation and health status among older adults, a population that has received relatively less scholarly attention. By highlighting the intersection of gender and social stratification, the findings demonstrate that sport participation in later life cannot be fully understood through material or socioeconomic conditions alone. Cultural expectations and the structural challenges associated with aging also play a critical role in shaping both sport participation and health.

Given these differences in types of sport participation, as well as patterns of subjective health, the findings highlight the need for policy interventions that reduce structural barriers faced by older adults. Although the Framework Act on Sports affirms the right of all citizens to enjoy sport, our results show that access remains unequal across gender and social strata. To address this gap, governments should expand support for low-cost, community-based programs that are accessible to older adults with limited economic resources; develop gender-inclusive initiatives that encourage broader participation across traditionally “masculine” and “feminine” sport domains; and offer flexible scheduling and neighbourhood-based options that accommodate the time constraints faced especially by older women. These measures would help translate the normative goals of sport equity into practice and promote healthier aging.

However, this study has several limitations. While it identified sport participation types across gender and social stratification, it did not fully address the psychological processes that may influence sport participation and health status among older adults in South Korea. Additionally, other important factors such as regional disparities in access to facilities and family caregiving responsibilities were not included in the analysis. Finally, the study relied on cross-sectional data, limiting the ability to draw conclusions about changes over time. Future research should adopt longitudinal designs and incorporate qualitative approaches to better understand the dynamic interplay between gender and social stratification in shaping sport participation and health status in later life.

## Figures and Tables

**Table 1:** Participants’ demographics.

Variables		Frequency	%
Gender	Male	834	46.9
	Female	945	53.1
Income	<2055 USD	938	52.7
	2055–2740 USD	359	20.2
	2740–3425 USD	214	12.0
	≥3425 USD	268	15.1
Education	Up to lower secondary	651	36.6
	Upper secondary	950	53.4
	Bachelor’s level or higher	178	10.0
Job	No occupation	912	51.3
	Blue-collar workers	517	29.1
	Sales & Service workers	281	15.8
	Office & Professionals	69	3.9
Sports	Walking	1115	62.7
	Gate ball	41	2.3
	Golf	56	3.1
	Aerobics & aquarobics	43	2.4
	Hiking	152	8.5
	Bodybuilding	69	3.9
	Swimming	76	4.3
	Yoga & Pilates	56	3.1
	Cycle	32	1.8
	Stretching exercises	90	5.1
	Table tennis	49	2.8
Total		1779	100

**Table 2:** Model summary resulting from the MCA.

Dimension	Cronbach’s Alpha	Variance Accounted for Total (Eigenvalue)	Inertia	% of Variance
1	0.915	3.191	0.798	79.770
2	0.744	2.262	0.566	56.561
Total		5.453	1.363	
Mean	0.844	2.727	0.682	68.166

**Table 3:** Cross-tab results of types of sports participation according to gender and social stratification.

		Income ^1^								Education ^2^						Job ^3^							
		Men				Women				Men			Women			Men				Women			
		a	b	c	d	a	b	c	d	1	2	3	1	2	3	A	B	C	D	A	B	C	D
Walking	C ^4^	295	93	54	46	376	118	65	68	184	253	51	290	315	22	218	212	47	11	410	112	98	7
	AR ^5^	4.2	−2.7	−2.3	−7.8	5.0	0.4	−0.8	−2.1	3.9	−4.4	−4.6	4.5	1.3	−4.1	4.0	−1.1	−6.2	−6.6	2.7	1.6	0.3	−1.0
Gateball	C	17	6	1	2	10	3	0	2	10	15	1	8	7	0	17	9	0	0	11	3	1	0
	AR	2.8	1.0	−0.9	−0.7	−0.7	−0.6	−1.7	−0.6	1.9	1.5	−1.1	−0.5	−1.5	−1.2	4.3	0.3	−1.8	−1.2	−1.0	−0.4	−1.4	−0.6
Golf	C	3	5	9	24	5	2	3	5	1	11	29	4	5	6	2	8	13	18	9	1	4	1
	AR	−3.2	−0.2	3.3	9.8	−3.4	−1.7	−0.3	0.5	−2.7	−1.1	13.7	−2.8	−3.1	3.1	−2.7	−1.1	4.7	12.8	−2.9	−2.0	−0.4	0.9
Aerobics, Aquarobics	C	2	0	0	0	20	8	9	4	0	2	0	13	26	2	1	1	0	0	33	4	4	0
	AR	−2.9	−2.2	−1.7	−2.0	2.5	1.8	4.1	0.6	−2.7	−3.3	−1.8	1.2	5.0	0.5	−2.6	−2.9	−1.8	−1.2	5.9	0.0	0.2	−0.6
Hiking	C	31	24	19	35	11	7	8	17	11	89	9	3	33	7	15	57	27	10	13	14	15	1
	AR	−0.9	2.6	3.7	7.2	−6.3	−2.4	−0.5	2.1	−2.5	9.5	−0.4	−6.4	−1.6	0.9	−2.4	5.7	5.3	2.6	−7.0	0.0	0.6	−0.2
Bodybuilding	C	18	10	9	11	9	5	2	5	9	29	10	3	15	3	17	14	12	5	10	4	6	1
	AR	0.6	1.3	2.6	2.5	−3.1	−0.9	−1.2	0.1	−0.2	3.1	2.6	−3.7	−1.0	0.5	1.8	0.1	3.4	2.0	−3.6	−1.0	0.0	0.6
Swimming	C	3	5	6	2	20	19	9	12	2	11	3	14	38	8	1	8	4	3	45	4	10	1
	AR	−4.1	−1.0	0.8	−1.8	−0.6	4.3	2.1	3.0	−2.9	−2.4	−1.0	−0.9	4.6	3.6	−3.7	−2.1	−0.6	0.4	4.6	−1.2	1.4	0.5
Yoga, Pilates	C	0	0	0	0	31	10	8	7	0	0	0	28	19	9	0	0	0	0	38	9	8	1
	AR	−4.2	−2.5	−1.9	−2.2	4.3	1.9	2.6	1.6	−3.1	−4.5	−2.0	5.0	1.1	5.4	−3.4	−3.8	−2.1	−1.4	5.3	1.8	1.5	0.9
Cycle	C	11	9	2	8	0	1	1	0	5	20	5	1	1	0	4	17	7	2	2	0	0	0
	AR	1.5	3.5	0.1	3.6	−3.7	−1.4	−0.7	−1.6	0.3	4.7	2.0	−2.7	−3.1	−1.1	−0.7	4.8	3.3	1.0	−3.4	−1.8	−1.8	−0.5
Stretching exercise	C	17	8	0	7	41	10	2	5	15	13	4	37	20	1	15	10	4	3	39	13	5	1
	AR	−1.0	−0.3	−2.4	−0.1	3.4	0.2	−1.6	−0.6	0.7	−2.6	−0.9	4.3	−1.1	−1.2	0.0	−2.1	−1.0	0.1	1.8	1.8	−1.1	0.4
Table tennis	C	16	15	4	7	2	1	3	1	12	23	7	1	5	1	9	17	13	3	3	0	3	1
	AR	1.6	5.0	0.7	1.7	−4.0	−1.9	0.0	−1.4	2.1	3.3	2.2	−3.5	−2.7	−0.5	0.3	2.6	5.3	1.2	−4.2	−2.3	−0.6	1.0

^1^ a: Less than 2055 USD/b: 2055–2740 USD)/c: 2740–3425 USD)/d: 3425 USD and above; ^2^ 1: Up to lower secondary/2: Upper secondary/3: Bachelor’s level or higher; ^3^ A: No occupation/B: Blue-collar workers/C: Sales & Service workers/D: Office & Professionals; ^4^ C, Count.; ^5^ AR, Adjusted Residual.

**Table 4:** Results of one-way ANOVA and Scheffé post hoc tests for subjective health status according to social stratification by gender.

			Subjective Health Status					
			Men			Women		
			*n*	M	SD	*n*	M	SD
Social stratification	Income	<2055 USD (a)	413	3.03	0.80	525	2.94	0.77
		2055–2740 USD (b)	175	3.45	0.68	184	3.28	0.65
		2740–3425 USD (c)	104	3.59	0.63	110	3.52	0.62
		≥3425 USD (d)	142	3.58	0.69	126	3.63	0.63
		F-value	31.739 ***			46.094 ***		
		post hoc	a < b, c, d			a < c, d		
	Education	Up to lower secondary (a)	249	2.91	0.81	402	2.90	0.75
		Upper secondary (b)	466	3.39	0.72	484	3.36	0.71
		Bachelor’s level or higher (c)	119	3.62	0.65	59	3.49	0.63
		F-value	50.147 ***			50.624 ***		
		post hoc	a < b < c			a < b, c		
	Occupation	No occupation (a)	299	2.91	0.82	613	3.07	0.76
		Blue-collar workers (b)	353	3.45	0.66	164	3.25	0.74
		Sales & service worker (c)	127	3.46	0.71	154	3.47	0.69
		Office & professionals (d)	55	3.78	0.60	14	3.43	0.76
		F-value	43.379 ***			13.376 ***		
		post hoc	a < b, c, d			a < c		

*** *p* < 0.001.

**Table 5:** Results of one-way ANOVA and Scheffé post hoc tests for subjective physical fitness according to social stratification by gender.

			Subjective Physical Fitness					
			Men			Women		
			*n*	M	SD	*n*	M	SD
Social stratification	Income	<2055 USD (a)	413	2.90	0.76	525	2.84	0.73
		2055–2740 USD (b)	175	3.27	0.71	184	3.14	0.71
		2740–3425 USD (c)	104	3.42	0.65	110	3.30	0.63
		≥3425 USD (d)	142	3.51	0.69	126	3.37	0.64
		F-value	34.565 ***			28.833 ***		
		post hoc	a < b, c, d			a < c, d		
	Education	Up to lower secondary (a)	249	2.80	0.80	402	2.77	0.73
		Upper secondary (b)	466	3.26	0.71	484	3.19	0.68
		Bachelor’s level or higher (c)	119	3.44	0.67	59	3.39	0.59
		F-value	42.578 ***			49.014 ***		
		post hoc	a < b < c			a < b, c		
	Occupation	No occupation (a)	299	2.77	0.75	613	2.93	0.73
		Blue-collar workers (b)	353	3.31	0.68	164	3.10	0.71
		Sales & service worker (c)	127	3.43	0.70	154	3.30	0.66
		Office & professionals (d)	55	3.55	0.69	14	3.36	0.63
		F-value	46.873 ***			12.893 ***		
		post hoc	a < b, c, d			a < c		

*** *p* < 0.001.

## Data Availability

The microdata from the 2024 Korea National Sports Participation Survey (KNSPS) are publicly available and can be downloaded without restriction from the Korea Institute of Sport Science (KISS) data portal. The dataset is accessible at: https://www.sports.re.kr/front/board/bs/boardView.do?board_seq=74&pageNo=1&menu_seq=865&con_seq=6288 (accessed on 25 April 2025).
